# Elevated asprosin levels in epicardial adipose tissue: Implications for coronary artery disease

**DOI:** 10.34172/jcvtr.026.33554

**Published:** 2026-03-30

**Authors:** Hossein Shateri, Babak Manafi, Amir Hossein Yazdi, Sayed Mostafa Hosseini

**Affiliations:** ^1^Student Research Committee, Baqiyatallah University of Medical Sciences, Tehran, Iran; ^2^Department of Surgery, Faculty of Medicine, Hamadan University of Medical Sciences, Hamadan, Iran; ^3^Clinical Research Development Unit Farshchian Heart Center, Department of Cardiology, School of Medicine, Hamadan University of Medical Sciences, Hamadan, Iran; ^4^Chemical Injuries Research Center, Systems Biology and Poisonings Institute, Baqiyatallah University of Medical Sciences, Tehran, Iran

**Keywords:** Coronary artery disease, Epicardial adipose tissue, Asprosin

## Abstract

**Introduction:**

This study investigates the role of the adipokine asprosin in coronary artery disease (CAD) by comparing its levels in epicardial adipose tissue (EAT) and serum between individuals with and without CAD. Asprosin is known to be involved in conditions such as insulin resistance and obesity, both of which are risk factors for CAD.

**Methods:**

The study included 25 CAD patients and 25 non-CAD patients undergoing open-heart surgery for valve issues. EAT samples were collected during surgery, and the mRNA levels of the FBN-1 gene were measured using qRT-PCR, while the protein levels of asprosin in both EAT and serum were determined using the ELISA method.

**Results:**

The results showed that the mRNA level of FBN-1 in the EAT of CAD patients was significantly higher than that in the control group (*P*<0.001). Additionally, asprosin protein levels were significantly elevated in both the EAT (*P*<0.003) and serum (*P*<0.027) of CAD patients compared to non-CAD individuals.

**Conclusion:**

These findings suggest that asprosin may play a critical role in the pathogenesis of CAD, particularly through its increased presence in EAT and serum. The study highlights the potential of asprosin as a biomarker for CAD, though further research is needed to fully understand its mechanisms in the progression of the disease.

## Introduction

 The primary cause of mortality in most advanced countries is coronary artery disease (CAD), which arises from the process of atherosclerosis in the coronary arteries. This process is influenced by various risk factors including, age, smoking, and obesity. The gradual accumulation of fatty substances in the vessel walls leads to the formation of atherosclerotic plaques, paving the way for vascular obstruction and clinical phenomena such as heart attacks, which pose a life-threatening risk to individuals. Statistics indicate that approximately one out of every four deaths is attributable to CAD.^1^

 Sedentary lifestyle, insufficient physical activity, and obesity have now become major risk factors for this disease.^1^ Adipose tissue dysfunction, characterized by enlarged adipocytes and macrophage infiltration, contributes significantly to the pathogenesis of atherosclerosis. This dysfunction triggers chronic inflammation within adipose tissue, reducing the secretion of protective adiponectin and increasing proinflammatory cytokines like IL-6, IL-8, and MCP-1. Additionally, the release of TNF-α from macrophages exacerbates local inflammation, impacting cardiovascular health and insulin sensitivity. Ectopic fat accumulation in various tissues further exacerbates insulin resistance and cardiovascular complications associated with metabolic syndrome.^2,3^

 Epicardial adipose tissue (EAT), which is the visceral fat around the heart, is a significant fat depot in the body. Previous studies indicate that ectopic fat accumulation and disruption in secretory substances also occur within it. The diverse range of bioactive molecules produced by EAT reflects its complex cellularity and interactions with neighboring structures, including cardiomyocytes and vascular cells, through paracrine or vasocrine signaling pathways. These interactions may influence inflammation, atherogenesis, and cellular cross talk within the cardiovascular system. Adipokines play an important role in the initiation and progression of CAD.^4-6^

 Asprosin is one of the recently discovered adipokines,^7^ and its levels have been found to increase in the circulation of patients with CAD. Moreover, its levels are significantly related to the severity of the disease in patients with unstable angina pectoris.^8,9^ An increase in the serum level of asprosin has been identified in some diseases such as T2DM,^10^ gestational diabetes,^11^ PCOS^12^ and non-alcoholic fatty liver disease.^13^ On the other hand, the protective role of asprosin on cardiomyocytes in response to hypoxia and its role in improving microvascular endothelial damage in the heart in diabetic rats suggest that the regulation of asprosin levels is important for heart health.^14^ Asprosin is a glucogenic hormone that regulates hepatic glucose release and is a cleavage product of the C-terminal protein profibrillin-1. It is also effective in stimulating appetite and storing fat.^7,15^ Asprosin is mainly synthesized by white adipocytes, but it is also expressed in other tissues.^7^ So far, no study has investigated the protein level of this adipokine in EAT. Based on this, the present study investigated its protein level in EAT and serum of CAD patients compared to non-CAD patients. This study aimed to investigate the expression of FBN-1 in EAT and evaluate its protein product, asprosin, as a potential biomarker in patients with and without CAD.

## Materials and Methods

###  Study Population

 This case-control study was conducted in the Farshchian Cardiovascular Subspecialty Medical Center (Hamadan-Iran) on patients who underwent open-heart surgery. The patients were divided into two groups: CAD group: including 25 patients with CAD who had more than 50% stenosis in at least one of the main coronary arteries and were candidates for selective coronary artery bypass grafting (CABG). Non-CAD group: including 25 patients who were candidates for open-heart surgery to replace the valve with a normal coronary anatomy (according to the angiography report, participants in the non-CAD group had less than 50% coronary artery stenosis and no clinical symptoms suggestive of CAD). The sample size (n = 50; 25 per group) was determined based on feasibility and the limited number of eligible patients undergoing open heart surgery during the study period. Although a formal power analysis was not performed, the selected sample size is consistent with previous pilot studies involving EAT sampling in the cardiovascular setting. Patients with kidney disease, liver disease, recent acute myocardial within the last month, any type of malignant disease, recent major abdominal surgery (within the last six months), diabetes mellitus, and heart failure in the final stage were excluded from the study. Demographic characteristics, anthropometric measures, risk factors, and medication use were recorded and clinical information was collected for each patient. This study was conducted in accordance with the ethical guidelines of the 2003 Declaration of Helsinki and the ethical standards of the National Research Committee. This study was approved by the Research Ethics Committee of Baqiyat Elah University of Medical Sciences (Tehran-Iran), and written informed consent was obtained from all participants.

###  Sample Collection 

 Prior to the commencement of open-heart surgery, under the supervision of a specialized surgeon, EAT (weighing between 300-500 mg) was extracted from the vicinity of the right coronary arteries of the study participants. The tissue samples were then cleaned with phosphate-buffered saline (PBS) solution and promptly preserved in liquid nitrogen, then stored at -80°C thereafter. Additionally, a 5 mL sample of venous blood was collected to measure the levels of fasting blood sugar (FBS), and serum triglycerides (TG), total cholesterol (TC), low-density lipoprotein cholesterol (LDL-C), and high-density lipoprotein cholesterol (HDL-C) using commercially available kits (Pars Azmoon, Tehran, Iran).

###  Determination of FBN-1 Gene Expression Levels by qRT -PCR

 The mRNA levels of FBN-1, the gene responsible for asprosin synthesis, were quantified in EAT using quantitative real-time polymerase chain reaction (qRT-PCR). Total RNA extraction from EAT samples was performed using RNX-Plus reagent (Sinagen, Iran) according to the manufacturer’s instructions. RNA purity and concentration were assessed using a NanoDrop^TM^ ND-2000 spectrophotometer (Thermo Fisher, USA). The absorbance ratio A260/A280 was in the range of 1.8 to 2.0, and the ratio A260/A230 was greater than 2.0, indicating adequate RNA purity. All samples were examined for RNA integrity by agarose gel electrophoresis, and 28S and 18S rRNA bands were observed (Supplementary Figure S1). cDNA synthesis was performed using the RevertAid First Strand cDNA Synthesis Kit (Thermo Fisher Scientific Inc., USA) and oligo(dT) primers; stem-loop or polyA tailing methods were not used. Each qPCR reaction was performed in a final volume of 20 μL containing the following: 10 μL of 2X SYBR Green Master Mix (Amplicon, Denmark), 2 μL of cDNA (equivalent to 50 ng RNA), 0.5 μL of each of the forward and reverse primers (Table 1) at 10 μM, and 7 μL of nuclease-free water. All reactions were performed in triplicate using a LightCycler® 96 instrument (Roche Life Science, Germany). To control for contamination, a no-template control (NTC) and a no-RT control were used to confirm the absence of genomic DNA contamination or other sources. The β-actin gene was used as a reference gene to normalize gene expression. The resulting qRT-PCR signals were validated by visualizing the bands on 2% agarose gel electrophoresis, compared against a 100 bp DNA ladder marker. The relative mRNA expression levels were determined relative to the expression of β-actin using the 2^-ΔCt^ method.

**Table 1:** Sequences of forward and reverse primers

**Gene names**		**Primer sequences**
*ACTB*	Forward	5′-ACAGAGCCTCGCCTTTGC-3′
	Reverse	5′-ATCACGCCCTGGTGCCT-3′
*FBN-1*	Forward	5′-GTACGAACACAGTCAGCAGTTA-3′
	Reverse	5′–TATCCTGGGCGGACATCTAT-3′

ACTB: b-actin, Fibrillin-1.

###  Determination of Asprosin Protein Levels in EAT and Serum by ELISA

 Protein extraction from approximately 100 mg of frozen EAT samples was performed using radioimmunoprecipitation assay (RIPA) lysis buffer (Santa Cruz Biotechnology, CA, USA). Briefly, the tissue samples were homogenized for 5 minutes on ice in 600 µl lysis buffer supplemented with 15 µl of protease inhibitors cocktail (Sigma Aldrich, Missouri, USA) and phosphatase inhibitors to yield total protein. Subsequently, the samples were shaken for 2 hours at 4°C, followed by centrifugation at 12,000 x g for 20 minutes at 4°C. The resulting supernatants were collected and stored at -20°C until further analysis. The total protein concentration was determined using the bicinchoninic acid protein assay (BCA) method.

 The expression levels of asprosin protein in both EAT and serum samples were assessed using the Human asprosin ELISA Kit (ZellBio GmbH, Germany) in accordance with the manufacturer’s instructions. Sample concentrations were calculated based on the standard curve and expressed as ng of total protein (ng/µg) in EAT and ng/mL in serum.

###  Statistical Analysis

 Statistical analyses were performed using SPSS software version 16.0 (SPSS Inc., Chicago, IL, USA) and GraphPad Prism software version 6.0 (Graph Pad Software, CA, USA). Differences between CAD and control patients were assessed using an independent samples T-test and the Mann-Whitney U test, depending on the normality of the variables. Chi-square analysis was employed to evaluate differences in qualitative variables between the two groups. Group comparisons of asprosin levels were adjusted for age, sex, and BMI using analysis of covariance (ANCOVA). Spearman’s correlation coefficients were calculated to determine correlations between variables. Results are presented as means ± standard deviation (SD) or mean ± standard error of the mean (SEM) as appropriate, with statistical significance defined as *P* < 0.05.

## Results

###  Demographic Characteristics, Anthropometric Measures, Clinical Data and Risk Factors

Table 2 presents a summary of the demographic characteristics, anthropometric measures, clinical data, and risk factors. No notable discrepancies were observed in age, body mass index (BMI), or gender distribution across the groups. While total cholesterol, triglyceride levels, and LDL cholesterol levels showed no significant difference between the groups, HDL cholesterol levels were notably lower in the CAD group compared to the control group (*P* = 0.001).

**Table 2:** Demographic characteristics, anthropometric measures, clinical data, risk factors and consumption of medication

	**CAD (n=25)**	**Non-CAD (n=25)**	***P*** ** value**
Demographic and anthropometric characteristics			
Age (years)	53.87 ± 4.95	52.45 ± 4.54	0.150
BMI (kg/m2)	26.07 ± 1.99	25.34 ± 1.48	0.220
Male sex (n, %)	13 (52.00%)	13 (52.00%)	0.611
Clinical data			
FBS (mg/dl)	101.25 ± 12.99	95.30 ± 11.12	0.164
TC (mg/dl)	149.02 ± 38.28	142.25 ± 38.37	0.120
TG (mg/dl)	136.64 ± 91.55	125.15 ± 88.38	0.080
HDL-C (mg/dl)	33.16 ± 09.99	42.12 ± 10.00	**0.001**
LDL-C (mg/dl)	106.01 ± 37.02	87.29 ± 36.28	0.210
SBP (mmHg)	115.66 ± 14.71	118.02 ± 8.18	0.374
DBP (mmHg)	71.24 ± 09.74	75.01 ± 10.10	0.605
LVEF (%)	47.99 ± 5.23	52.01 ± 7.31	0.105
Risk factors			
HLP (n, %)	21 (84.00%)	2 (8.00%)	**0.002**
HTN (n, %)	16 (64.00%)	4 (16.00%)	**0.005**
FH (n, %)	18 (72.00%)	4 (16.00%)	**0.023**
Current smoker (n, %)	17 (68.00%)	11 (44.00%)	0.100

Data are presented as mean ± SD and n (%). BMI: body mass index, CAD: coronary artery disease, DBP: diastolic blood pressure, FBS: fasting Blood Sugar, FH: family history of CAD, HDL-C: high-density lipoprotein cholesterol, HLP: hyperlipidemia, HTN: hypertension, LDL-C: low-density lipoprotein cholesterol, LVEF: left ventricular ejection fraction, SBP: systolic blood pressure, TC: total cholesterol, TG: Triglyceride.

###  Changes in mRNA Levels of FBN-1

**Figure 1:**
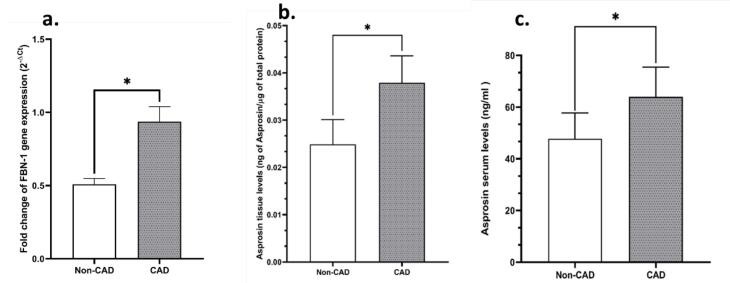
Expression levels of FBN-1 mRNA and Asprosin protein in CAD and non-CAD groups. a) FBN-1 mRNA levels in EAT in the CAD group were significantly higher than those in the non-CAD group (*P* = 0.010), after adjustment for age, sex, and BMI. b) Asprosin protein expression in EAT was significantly increased in CAD patients (*P* = 0.003), after adjustment for similar variables. c) Serum Asprosin protein levels were also significantly higher in CAD patients than in non-CAD subjects (*P* = 0.027). Data are presented as mean ± standard error (SEM). Asterisks indicate statistically significant differences between groups

 The investigations revealed that changes in mRNA levels of FBN-1 exhibited a non-normal distribution. Consequently, the Mann-Whitney U test was used to compare the alterations in mRNA levels of FBN-1 were compared between the two groups. As depicted in Figure 1a, the results demonstrated a significant increase in mRNA levels of FBN-1 in the CAD group compared to the non-CAD group (*P* < 0.001). This significant increase remained after adjusting for other variables including, age, gender, and BMI (*P* = 0.010).

###  Changes in Tissue and Serum Levels of Asprosin

 As shown in Figure 1b, analysis of asprosin protein level in epicardial fat tissue showed that the amount of asprosin in epicardial fat tissue of patients with CAD is significantly higher than that in non-CAD subjects. Also, Figure 1C illustrates that serum levels of asprosin were higher in patients compared to non-CAD subjects. Due to the possible effect of age, sex, and BMI factors, the results were adjusted for the mentioned variables, and the changes remained significant after adjusting.

## Discussion

 Coronary artery disease (CAD) accounts for a significant proportion of global cardiovascular disease-related deaths, with substantial regional variations. The increasing prevalence of risk factors such as obesity, smoking, and sedentary lifestyles contributes to the rising incidence of CAD.^16^ The pathophysiology of CAD involves several interrelated mechanisms. The disease primarily results from atherosclerosis, characterized by the buildup of fatty deposits (plaques) in the coronary arteries. This process is driven by a combination of risk factors, including hypertension, dyslipidemia, and diabetes mellitus, leading to endothelial dysfunction and chronic inflammation.^1^

 Epicardial adipose tissue (EAT) is a visceral fat depot located between the myocardium and visceral pericardium and is in close contact with the coronary arteries. Because of this anatomical proximity, EAT can influence coronary vascular physiology through paracrine and vasocrine signaling. The metabolic activity of this tissue and the secretion of inflammatory mediators have made it a subject of interest in the study of atherosclerosis and CAD.^4,5^ it has been shown that in patients with CAD, EAT secretes higher levels of inflammatory chemokines and cytokines like MCP-1, IL-1β, IL-6, and TNF-α, compared to subcutaneous fat (SAT), as well as increased infiltration of immune cells like macrophages. This suggests EAT’s role in vascular inflammation, plaque instability, and new blood vessel formation. Additionally, EAT in CAD patients has elevated reactive oxygen species (ROS) and lower antioxidant enzymes like catalase, contributing to oxidative stress. EAT may also promote early atherosclerosis by increasing monocyte migration to arterial walls through molecules like VCAM-1. Furthermore, EAT produces various adipokines that can affect coronary artery walls, with some, like adiponectin, having protective effects, while others, like visfatin and resistin, promote disease progression.

 The study emphasizes EAT’s role in CAD pathogenesis, focusing on its secretion of adipokines under disease conditions, and investigates a newly discovered adipokine potentially linked to EAT.

 Asprosin is a newly identified protein hormone derived from profibrillin-1, a protein encoded by the FBN-1 gene.^7,15^ FBN-1 is an extracellular matrix protein involved in maintaining vascular structure and is expressed in connective tissues including arteries and adipose tissue; adipose tissue is the main source of asprosin in the circulation.^7,17^

 In the context of CAD, FBN-1 plays a multifaceted role in pathological processes, particularly those related to vascular remodeling and inflammation.^17,18^ FBN-1 is essential for the formation of microfibrils, which provide structural support and elasticity to blood vessels.^17^ Abnormalities in FBN-1 can lead to compromised vascular integrity, contributing to the development of atherosclerosis—a hallmark of CAD.^17,19^ Increased levels of FBN-1 have been associated with vascular remodeling in CAD, affecting plaque stability and potentially leading to acute coronary events such as heart attacks.^20^

 The present study is the first to explore the expression of the FBN-1 gene in EAT and to assess its protein product, asprosin, in individuals with and without CAD. The findings revealed that FBN-1 expression was significantly higher in the EAT of CAD patients compared to those without CAD. This aligns with previous research suggesting that FBN-1 contributes to vascular changes in CAD, influencing both the inflammatory response within the vascular wall and the structural integrity of coronary arteries.^17^ Additionally, mutations in FBN-1 are linked to cardiovascular complications in Marfan syndrome, further emphasizing its critical role in cardiovascular health.^19^ These results underscore the importance of FBN-1 and asprosin in the pathology of CAD, highlighting their potential as targets for therapeutic intervention.

 In another finding of the present study, it was shown that the protein level of asprosin in EAT and serum of people with CAD was significantly increased compared to non-CAD subjects. Little is known about the association of asprosin with CAD. So far, no study has been conducted on the expression of pro-asprosin in EAT, and this study is the first in this field. Previous studies have shown that asprosin is mainly synthesized by white adipocytes, but it is also expressed in tissues, including the placenta in humans and surface epithelial cells of the gastric fundus, renal cortical distal tubules, and cardiac cardiomyocytes in mice.^14,21,22^ In the study of Moradi et al^9^ in 2021, the serum level of asprosin, along with IL-6, TNF-α, and adiponectin levels, were evaluated in 88 patients with CAD and 88 healthy non-CAD subjects. Their results showed that serum asprosin was higher in CAD patients compared to non-CAD patients. They also showed that serum asprosin level was directly correlated with BMI, FBG, HOMA-IR, TG, and TC. They proposed the possible relationship between serum asprosin and the pathogenesis of CAD.^9^

 Also, in a similar study, Güven et al in 2021 studied the plasma level of asprosin in patients with CAD who underwent routine coronary angiography for the first time. Patients were divided into four groups: including 20 people as a control group, a group of patients with CAD who had one coronary lesion, a group of patients with CAD who had two coronary lesions, and a group of patients with CAD who had multiple coronary lesions. They showed that the amount of asprosin in the group with multiple coronary lesions was significantly higher than in other groups. They concluded that an increase in serum asprosin level along with an increase in the number of coronary vessels can be a marker in diagnosing and determining the severity of CAD.^8^ In the present study, this issue was emphasized and it was shown that the serum level of asprosin is higher in CAD patients. Interestingly, our results are consistent with those of Acara et al,^23^ who reported that asprosin levels correlate with disease severity in patients with unstable angina, suggesting asprosin’s involvement in CAD progression. However, while Wen et al^14^ demonstrated a protective role for asprosin against adverse events in dilated cardiomyopathy, the elevated asprosin levels in CAD patients observed in this study indicate that its role may vary based on disease type or severity. The contradictory results between these studies highlight the complexity of asprosin’s influence on cardiovascular health, suggesting that asprosin may exert different effects depending on disease state, patient population, or study methodology, including the ELISA kits used for measurement.

 The observed increase in asprosin levels in both EAT tissue and serum of patients with CAD suggests the potential of this hormone as a novel biomarker for coronary artery disease. Although the current findings are preliminary, they suggest that asprosin may be a marker not only of the presence of the disease but also of its severity, especially given its association with inflammatory and metabolic markers. In clinical practice, asprosin may be useful in the early identification of at-risk individuals, particularly those undergoing cardiac evaluation and not yet exhibiting overt clinical symptoms. Measurement of serum asprosin levels may also be useful in stratifying CAD patients based on risk or plaque burden, complementing current diagnostic tools. However, prospective studies on a larger scale are needed to confirm its diagnostic and prognostic validity and to allow it to be incorporated into routine clinical assessments.

 Although FBN-1 is known as the precursor of asprosin, and both molecules were found to be elevated in EAT of CAD patients, the mechanistic pathway linking their expression in this specific fat depot remains unclear. Future studies should investigate whether upregulation of FBN-1 directly leads to increased asprosin production in EAT and how this may mechanistically contribute to coronary atherosclerosis.

 This study has several limitations. First, its cross-sectional design limits the ability to draw causal conclusions between high asprosin levels and the progression of CAD. Second, the relatively small sample size, although consistent with previous exploratory studies, limits the generalizability of the findings. Third, the mechanistic pathways of asprosin regulation in EAT and its direct impact on coronary artery pathology were not investigated, and longitudinal follow-up was not performed to assess prognostic implications. Future studies should include larger samples and experimental approaches to confirm these associations and clarify the functional role of asprosin in cardiovascular disease.

## Conclusion

 The results of this research showed that asprosin levels are significantly higher in the epicardial fat tissue and serum of people with CAD compared to non-CAD subjects. This demonstrates the importance of this new adipokine in investigating and understanding the pathways of CAD pathogenesis. However, more studies are needed to elucidate the mechanisms of this hormone’s effect. Future studies should explore the mechanistic role of FBN-1 and asprosin in EAT, particularly in relation to inflammatory signaling and plaque vulnerability in CAD.

## Competing Interests

 The authors have no relevant financial or non-financial interests to disclose.

## Ethical Approval

 This study was approved by the Research Ethics Committee of Baqiyat Elah University of Medical Sciences (approval ID: IR.BMSU.BAQ.REC.1403.018).

## Supplementary Files

Supplementary file 1 contains Figure S1.
