# Supplementary material for: Elevated asprosin levels in epicardial adipose tissue: Implications for coronary artery disease
Source: J Cardiovasc Thorac Res. 2026 Mar 30;18(1):32–7. doi: 10.34172/jcvtr.026.33554 (PMC13309332; doi:10.34172/jcvtr.026.33554)
Supplement: Supplementary file 1 — contains Figure S1. [file jcvtr-18-32-s001.pdf]

J Cardiovasc Thorac Res. 2026;18(1):32-S1
doi:10.34172/jcvtr.026.33554
http://jcvtr.tbzmed.ac.ir

1. **Supplementary File 1 - Signed Submission Checklist:**
   Please find the scanned copy of the signed checklist attached below.

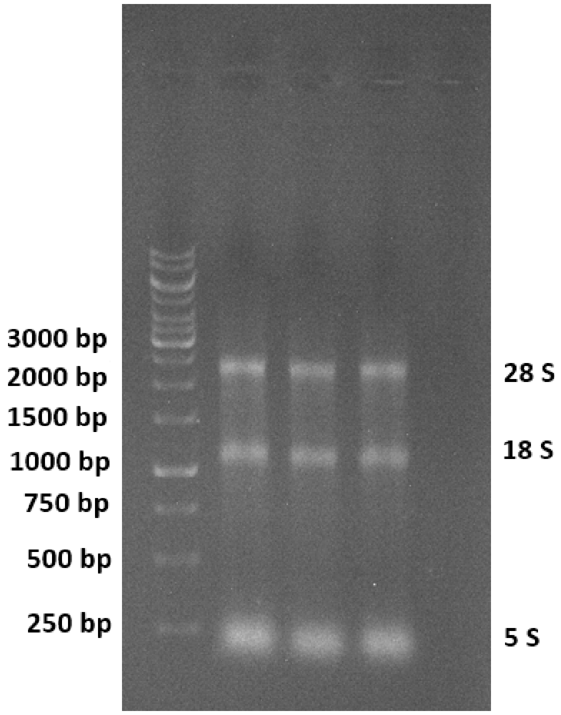

Supplementary Figure 1. examination of RNA integrity by agarose gel electrophoresis.
